# The Development of a Proactive Burnout Prevention Inventory: How Employees Can Contribute to Reduce Burnout Risks

**DOI:** 10.3390/ijerph17051711

**Published:** 2020-03-05

**Authors:** Madelon C. B. Otto, Joris Van Ruysseveldt, Nicole Hoefsmit, Karen Van Dam

**Affiliations:** Department of Psychology, Open University of the Netherlands, 6419 AT Heerlen, The Netherlands; joris.vanruysseveldt@ou.nl (J.V.R.); nicole.hoefsmit@ou.nl (N.H.); karen.vandam@ou.nl (K.V.D.)

**Keywords:** proactive behavior, burnout, prevention, inventory, scale development

## Abstract

Proactive burnout prevention refers to a set of proactive behaviors employees may engage in to prevent burnout. Findings of a previous exploratory qualitative study indicated that employees who had to deal with high demands engaged in specific proactive behaviors in the work, home, and personal domain in order to prevent burnout. To further examine proactive burnout prevention in longitudinal quantitative research and to be able to investigate its effectiveness, an inventory for assessing these kinds of behaviors is necessary. The goal of this study was twofold: 1) to develop an inventory to assess employees’ proactive burnout prevention behaviors and examine its factorial validity, 2) to explore the broader nomological network of proactive burnout prevention behaviors by examining its convergent, discriminant, and predictive validity. A two-wave longitudinal survey (T1: *N* = 343; T2: *N* = 201) was conducted. The results of exploratory and confirmatory factor analysis showed that proactive burnout prevention can be reliably assessed with 40 items that load on 12 factors, indicating 12 separate proactive burnout prevention behaviors. Moreover, exploration of the convergent, discriminant, and predictive validity of the proactive burnout prevention inventory showed promising results, as expected relationships were confirmed for most behaviors. Further research is needed to substantiate the findings and examine the effectiveness of proactive burnout prevention.

## 1. Introduction

Ample research has established that employees who are confronted with high job demands together with low job resources are at risk of burnout (e.g., [1,2]). These studies often suggest practical recommendations informing organizations which actions they can take to prevent or reduce burnout risks. However, the actions that employees themselves can take have received less research attention [3]. This is remarkable, since burnout develops in an interchange between employees and their work environment [2,4], indicating that both the employer and the employee can intervene to prevent burnout. In a position study, Demerouti [3] presented strategies individuals may use to reduce the detrimental effects of job stressors, and concludes that knowledge on these bottom-up interventions may be vital to complement top-down organization initiated interventions to prevent burnout. Still, as Demerouti [3] also stated, more research is needed to uncover the possible actions employees may undertake in an attempt to avoid burnout.

Burnout has recently been defined as ‘a work-related state of exhaustion that occurs among employees, which is characterized by extreme tiredness, reduced ability to regulate cognitive and emotional processes, and mental distancing’ [2]. These key symptoms may be complemented by secondary symptoms, such as feelings of depression, and behavioral and psychosomatic complaints of strain [2]. Since the consequences of burnout on individuals’ physical health and psychological wellbeing can be extremely severe, including coronary heart disease, gastrointestinal issues, prolonged fatigue, insomnia, mental health issues [5], employees have a clear interest in proactively engaging in actions to prevent burnout.

According to the conservation of resources (COR) theory [6] and the job demands-resources (JD-R) model [7], psychological stress occurs when resources (e.g., social support, autonomy) become insufficient to meet (high) demands (e.g., workload, time pressure), which ultimately may lead to resource depletion and the development of burnout when resources are not timely replenished. To prevent stress, and the development of burnout, employees could therefore take initiative and proactively try to avoid resource depletion, by increasing resources and/or reducing demands.

Reviews of burnout prevention programs (e.g., interventions aimed at the development of knowledge and work-related skills, interventions based on cognitive behavioral techniques), found that these interventions have lasting, albeit small effects, therefore new types of interventions to prevent burnout are required [8,9]. Findings show that burnout prevention interventions have been mostly employer-initiated [8,9]. These interventions focus on how individuals cope with workplace stressors or how organizational factors that trigger burnout should be addressed [8,9]. Burnout prevention programs have seldom included factors beyond the work environment [8,9]. Although study findings have indicated that non-work factors, such as personal resources and home demands, can also influence the development of burnout, few studies have included these aspects [10]. Burnout prevention programs may be more effective by not only focusing on employer-initiated actions in the workplace but including employee-initiated actions and factors outside the work domain as well.

Proactive behaviors are self-initiated, anticipatory actions aimed at changing oneself and/or the situation to bring about an improved future [11]. Self-initiated behaviors of employees have become increasingly important for the success of contemporary organizations, as they are seen as the driving force for innovation and adaption to operate effectively in today’s uncertain and complex work environment [11]. Various proactive concepts, such as feedback-seeking behavior and individual innovation, have been studied, and have been found to be positively related to organizational outcomes, such as job performance and organizational effectiveness (e.g., [12,13]). In relation to burnout, proactive behaviors, such as voice [14] and job crafting [15], have been linked to reduced levels of burnout [16,17]. These findings indicate that proactive behaviors can be effective in the prevention of burnout.

Proactive burnout prevention is defined as self-initiated, change-oriented actions of employees aimed at the prevention of burnout [18]. Findings of an exploratory qualitative study that was performed as a first step to investigate proactive behaviors of employees in the prevention of burnout (henceforth referred to as the “exploratory qualitative study into proactive burnout prevention”; ESP), showed that employees who encounter aversive working conditions, may indeed engage in specific behaviors aimed at increasing resources and/or reducing demands in order to prevent burnout [18]. These findings corroborate with Parker et al.’s [19] notion that stressors can prompt proactive behaviors targeted at a decrease in the discrepancy between a current and desired situation. Participants reported that (high) demands in and beyond the workplace have led them to take proactive actions in the work, home and personal domain simultaneously. In order to investigate the effectiveness of employees’ proactive burnout prevention behaviors in (longitudinal) quantitative research, an inventory is needed to assess these kinds of behaviors.

The goal of the present research was twofold: (1) to develop an inventory that assesses employees’ proactive burnout prevention behaviors and examine its factorial validity; (2) to explore the broader nomological network of proactive burnout prevention by examining the association of these behaviors with related constructs and wellbeing.

This study contributes to the scientific knowledge on proactive behaviors and the prevention of burnout. In addition, it enables further investigation of the antecedents, outcomes, and boundary conditions of proactive burnout prevention. If research demonstrates that specific kinds of burnout prevention behaviors can be effective in avoiding burnout, this knowledge can serve as a base for the development and evaluation of an intervention that promotes these self-initiated behaviors.

## 2. Study 1: Inventory Development and Explorative Testing

The goal of the first study was to develop an inventory that assesses employees’ proactive burnout prevention behaviors and to examine its factorial validity and internal consistency. First, the inventory development is described, and then the results of the exploratory testing are presented.

### 2.1. Method

#### 2.1.1. Inventory Development

The present study builds upon the findings of the ESP [18] by using its outcomes as a basis for developing an inventory to assess specific kinds of proactive burnout prevention behaviors. Based on the findings of this previous study and the burnout literature (e.g., [4,6,20]), we propose that proactive burnout prevention captures 12 specific proactive behaviors that cover work-, home- and person-oriented actions, and aim at both demands and resources [18] (see Table 1 and Supplementary Materials).

The inventory consists of 12 scales measuring each of the proactive burnout prevention behaviors identified in the ESP [18]. Where possible, items for each scale were based on or adapted from existing validated instruments. If necessary, items of existing instruments were adapted to reflect self-initiated and change-oriented actions of employees to ascertain that all items were formulated as to effectively assess proactive behavior [17]. In case no existing validated scale could be found to match one of the 12 identified behaviors, items were developed by the researchers based on results of the ESP [18] taking into account the guidelines set for item wording by Hinkin [34]. To ensure adequate internal consistency, at least three items per scale were generated for each proactive action and all items were scored on a five-point Likert scale ranging from 1 (*never*) to 5 (*always*) [34]. To further assure content validity the proposed definition of proactive burnout prevention and the constructed items were thoroughly reviewed in several discussions within the research team, that has extensive experience and knowledge on the research topic. If items of the selected validated scales were not available in the Dutch language, they were translated and back translated by the researchers.

In total 56 items were generated that reflected the 12 proactive burnout prevention behaviors. Table 1 shows the number of items per proactive burnout prevention behavior and their source.

*Increasing*/*maintaining job control*. Five items were adapted from a job control measure by Zijlstra, Den Hoedt, and De Vries [21] to measure increasing/maintaining job control.

*Increasing*/*maintaining supervisor social support*. For actions aimed at increasing/maintaining supervisor social support, three items were adapted from the ‘relation with supervisor’-scale of the Dutch Questionnaire on the Experience and Evaluation of Work (QEEW) [22].

*Increasing*/*maintaining co-worker social support*. Four of the five items that were used to assess increasing/maintaining co-worker social support were adapted from a social support scale used by Peeters, Buunk, and Schaufeli [23] which is based on the informational and instrumental dimensions of social support distinguished by House [24]. A fifth item originated from the Dutch QEEW [22] regarding actively working on the overall relationship with co-workers.

*Feedback seeking*. A Dutch translation of the existing scale (three items) by Ashford [25] was used to measure feedback seeking.

*Seeking*/*performing tasks that energize*. Four items from the job crafting for learning scale were used to assess seeking/performing tasks that energize [26].

*Reducing hindering job demands*. Reducing hindering job demands was assessed using the following eight items: two items of the decreasing hindering job demands dimension of the job crafting scale by Nielsen and Abildgaard [27] were translated into Dutch, four items of the decreasing hindering job demands dimension of the Dutch job crafting scale by Tims et al. [35], and two items generated by the researchers based on findings of the ESP.

*Increasing*/*maintaining home autonomy*. Three items were adapted from Demerouti, Bakker, and Voydanoff [29] to measure increasing/maintaining home autonomy.

*Increasing*/*maintaining home social support*. To assess home social support, five items were generated, mirroring the items used for co-worker social support, as has been done in previous studies that assessed home social support [23,31].

*Reducing work-home conflict*. Seven items were generated to assess reducing work-home conflict. Two items were based on the work-life crafting scale developed by Peeters and Demerouti [33]; three of the items from the psychological detachment scale of the recovery experience questionnaire by Sonnentag and Fritz [32] were adapted and included; two items were developed by the researchers themselves, based on the findings of the ESP.

*Improving*/*maintaining physical health*. Four items were generated by the researchers to assess improving/maintaining physical health based on the self-initiated actions employees mentioned to undertake in the ESP.

*Improving*/*maintaining psychological wellbeing*. The researchers generated five items to assess the proactive conservation of psychological wellbeing based on the proactive actions participants mentioned in the ESP.

*Engaging in relaxing activities*. Three items of the relaxation scale from the recovery experience questionnaire of Sonnentag and Fritz [32] were adapted to assess how often employees proactively relax to prevent burnout.

#### 2.1.2. Explorative testing

A two-wave (longitudinal) research design was used to examine the inventory’s factorial validity. Participants were asked twice to complete an online survey with an interval of one month. Approval for this research was obtained from an internal academic ethical committee, implying that research participants were treated in accordance with the ethical guidelines set out by the Declaration of Helsinki [36].

#### 2.1.3. Participants and Procedure

Participants were recruited through several channels in the Netherlands. Employees of a Dutch branch of an international charity organization (*N* = 110) and an organization for vocational rehabilitation services (*N* = 180) were asked by their employer to participate voluntarily in this study. In addition, a request for participation in this research was published in newsletters to (former) students and employees of a university in the Netherlands and on the internal and external websites of this university. Participants had to meet the following inclusion criteria: employees who are 18 years or older, and not meet the exclusion criteria: employees who are on long-term (6 weeks or more) sick leave at the time of the study. The employees of the two participating organizations received an email from their employer containing a link to the online survey. The newsletters with the request for participation to the (former) students and employees of the university also contained a link to the online survey. After clicking on the link, the participants first received information on the research goals and procedure, a notification that participation is voluntary and can be terminated at any time during the research, and details on the storage of data (in line with the General Data Protection Regulation [37]). Before they actually could start answering the questions, their formal consent for participation was asked. Participants were asked to fill out the same survey twice, with an interval of approximately one month. As an incentive, participants were offered to receive a personal burn-out risk profile put together by the researchers, after they completed the survey on both occasions.

At T1 the online survey was fully completed by 343 participants. The overall response rate could not be established, since it was unknown how many people visited the website of the university and thus had access to our research. The average age was 43.6 years (*SD* = 11.2) and 74.6 percent were female. The educational level of the participants was relatively high, with 38.8 percent higher vocational education and 49.3 percent university degree. Participants worked on average 34.5 hours per week (*SD* = 8.9) in a range of industries, including education (23.0%), healthcare (17.2%), government (12.5%), charity (9.9%), reintegration services (9.0%), business/financial services (6.7%), IT (3.5%), or other sectors such as manufacturing, transport, and retail (18.2%). Of the 343 participants at T1, 201 also fully completed the online survey at T2. Analysis testing for systematic dropout from those who only responded at T1 (*N* = 142) to those who responded both times (*N* = 201), revealed no significant differences in terms of gender, education level, industry, and working hours, but did show a significant difference in age. The average age of those who only responded at T1 was 41.9 years (*SD* = 11.1), and the average age of those who responded at both times was 44.8 (*SD* = 11.1). However, no significant differences between the two groups were found on proactive burnout prevention behaviors and well-being outcomes.

#### 2.1.4. Analysis

Exploratory factor analysis (EFA) and confirmatory factor analysis (CFA) were performed on the items that were intended to assess the 12 proactive burnout prevention behaviors in order to examine factorial validity. Internal consistency was investigated by testing for internal consistency reliability using Cronbach’s alpha. The statistical software program SPSS 24 was used to perform the EFA and examine internal consistency, and the CFA was conducted using AMOS 24. In order to use two separate unique samples (e.g., no overlap in participants) for the EFA and CFA, T1 data from the participants who completed the online survey at T1 only (Sample 1; *N* = 142) were used to perform the EFA and assess internal consistency reliability, and T1 data from the participants who completed the online survey both at T1 and T2 (Sample 2; *N* = 201) were used to perform the CFA.

EFA (principal component analysis; PCA) with Promax rotation was performed to investigate whether the 12 behaviors representing proactive burnout prevention could be meaningfully distinguished from each other. Therefore, twelve components were extracted and the following criteria were used to evaluate the outcomes. Factors that loaded 0.35 or higher on the intended factor were retained [38]. Items were deleted if they loaded 0.35 or higher on another factor than the expected one, or if they did not correlate with at least two others. The remaining factors were tested for internal consistency (Cronbach’s alpha > 0.70; [39]) and items were excluded to obtain item reduction, if this would not harm the content validity and led to an improvement of the internal consistency. An exception was made if this meant that only two items would remain in a factor [34]. After removal of items, PCA was repeated on the new datasets until all criteria were satisfied.

CFA was performed to examine whether the 12-factor structure could be reliably replicated using a new sample. Latent variable structural equation modelling (SEM) with maximum likelihood estimation was used to conduct CFA. Three nested models were investigated. Model 1 was a correlated 12 factor model containing one latent factor for each of the 12 proactive burnout prevention behaviors, whereas each item loaded on the proactive behavior it represents. Alternatively, in Model 2 all items loaded on one latent factor, representing proactive burnout prevention behavior in general. Finally, in Model 3 the latent factors representing each of the 12 proactive burnout prevention behaviors loaded on a single second order factor. Multiple fit indices were used to evaluate model fit [40]: the model chi-square with degrees of freedom and probability level, the Tucker-Lewis Index (TLI ≥ 0.90), the comparative fit index (CFI ≥ 0.90), the incremental fit index (IFI ≥ 0.90), the root mean square errors of approximation (RMSEA < 0.08), and the standardized root mean square residual (SRMR < 0.08).

### 2.2. Results

#### 2.2.1. Exploratory Factor Analysis

Before performing the PCA, Bartlett’s test of sphericity and the Kaiser-Meyer-Olkin test were performed to establish whether the data were appropriate to perform this analysis. Bartlett’s test of sphericity was significant (*χ2* (1540) = 4956.15, *p* < 0.001), indicating that the use of the factor analytic model on this set of data was appropriate. The Kaiser-Meyer-Olkin measure of sampling adequacy suggested that the strength of the relationships among variables was high (KMO = 0.74), it was therefore acceptable to continue the analysis. Based on the set criteria nine of the initial 56 items were deleted after performing the initial PCA. The remaining factors were tested for internal consistency. This resulted in excluding three additional items. A second and third PCA were performed on the remaining items (resulting in excluding four additional items), before the final factor solution satisfied all specified criteria. This final solution with 40 items showed a clear 12-factor structure with all items loading on their intended factor, explaining 76.0 percent of variance in the items. Cronbach’s alpha’s of the 12 subscales ranged from 0.70 to 0.92, which is in line with the required 0.70 [39]. The items, means, standard deviations, Cronbach’s alphas, factor loadings of the pattern matrix, and factor intercorrelations are described in Table 2. In sum, the results indicate that all 12 previously identified behaviors [18] are included and represented in the proactive burnout prevention inventory. The scales possess satisfactory internal consistency reliability.

#### 2.2.2. Confirmatory Factor Analysis

We performed CFA to examine whether the 12-factor structure could be reliably replicated using a new sample. Model 1, representing the correlated 12 factor model containing one latent factor for each of the 12 proactive burnout prevention behaviors, showed good fit (*χ2* = 870.75, *df* = 666, *p* < 0.001; TLI = 0.94, CFI = 0.95, IFI = 0.95, RMSEA = 0.04, SRMR = 0.06). Moreover, all items loaded significantly on the latent variables, with factor loadings ranging from 0.51 to 0.94 (all *p*-values < 0.001). The two alternative models showed (very) poor fit. This was both applicable to the one latent factor Model 2 (*χ2* = 6927.03, *df* = 1180, *p* < 0.001; TLI = 0.26, CFI = 0.29, IFI = 0.29, RMSEA = 0.12, SRMR = 0.15) and to Model 3 (*χ2* = 2323,49, *df* = 1154, *p* < 0.001; TLI = 0.85, CFI = 0.86, IFI = 0.86, RMSEA = 0.06, SRMR = 0.10) which contained one second order factor on which all 12 proactive burnout prevention behaviors loaded. Consequently, results of the CFA confirmed the 12- factor structure.

Based on the results of the EFA and CFA it can be concluded that the 12-factor structure of the proactive burnout prevention inventory was empirically supported.

## 3. Study 2: Nomological Network

The second goal of this research was to gain insight into the wider nomological network of proactive burnout prevention by exploring its convergent, discriminant, and predictive validity.

Convergent validity refers to the extent to which two constructs that are theoretically related indeed converge as evidenced in a positive correlation between these constructs [39]. To demonstrate the convergent validity of proactive burnout prevention, the extent to which its behaviors were associated with proactive personality was examined. Individuals with a proactive personality have been found to be inclined to engage in proactive behavior [41]. It is therefore expected that employees with a proactive personality will undertake action and make changes to prevent burnout.

To establish discriminant validity the construct under research should show a non-significant or weak correlation with theoretically different constructs [39]. Generalized compliance was included in this study to explore the discriminant validity of proactive burnout prevention. Generalized compliance is a major aspect of organizational citizenship behavior and refers to strict adherence to internal rules and regulations [42,43]. Generalized compliance is similar to proactive burnout prevention, in that it also indicates a form of extra-role behavior, in which employees act beyond formal role requirements. However, it differs from proactive burnout prevention in that it is a more pro-social form of employee behavior that is indirectly helpful to others but is not aimed at (actively) changing oneself or the situation to prevent burnout. It was therefore expected that generalized compliance can be differentiated from proactive burnout prevention behaviors.

To further investigate the nomological network of the inventory, the predictive validity was examined. The goal of proactive burnout prevention is to avoid burnout. It was therefore expected that proactive burnout prevention behaviors were negatively related to burnout complaints. In contrast, proactive burnout prevention is aimed at conserving resources which may promote employees’ goal achievement and enhance feelings of fulfilment and commitment regarding work [44]. Since work engagement refers to a positive work-related mind-set [45], a positive relationship is therefore expected between proactive burnout prevention behaviors and work engagement.

### 3.1. Method

#### 3.1.1. Participants and Procedure

Convergent and discriminant validity were examined using both Sample 1 and Sample 2 data collected at T1 (*N* = 343). Predictive validity was investigated by exploring the lagged effect of proactive burnout prevention behaviors on burnout using Sample 2 data gathered at T1 and T2 (*N* = 201).

#### 3.1.2. Measures

*Proactive personality* was measured using the 6-item version of Bateman and Crant’s [41] proactive personality scale (α = 0.79), validated by Claes, Beheydt, and Lemmens [46]. An example item is: “*If I see something I don’t like, I fix it*”. Response categories ranged from 1 (*strongly disagree*) to 5 (*strongly agree*).

*Generalized compliance* was measured using four items of the measure developed by Smith, Organ, and Near [42] (α = 0.76). An example of an item is: “*In general, I try to keep good attendance at work*”. Response categories ranged from 1 (*strongly disagree*) to 5 (*strongly agree*).

*Burnout* was measured using the 23 item Burnout Assessment Tool [2] (α = 0.94). An example of an item is: “*At work, I feel mentally exhausted*”. Response categories ranged from 1 (*never*) to 5 (*always*).

*Work engagement* was assessed using the short version (nine items) of the Utrecht Work Engagement Scale (UWES; [47]) (α = 0.95). An example of an item is: “*My job inspires me*”. Response categories ranged from 1 (*never*) to 7 (*always*).

#### 3.1.3. Analysis

Pearson correlation analyses were performed using the statistical software program SPSS 24 to explore the convergent, discriminant, and predictive validity of the proactive burnout prevention inventory.

### 3.2. Results

Table 3 presents the means, standard deviations, Cronbach’s alphas and correlations of the variables. Eight of the 12 proactive burnout prevention behaviors showed a significant positive relationship with proactive personality: increasing/maintaining job control (*r* = 0.18, *p* < 0.01), increasing maintaining supervisor social support (*r* = 0.27, *p* < 0.01), increasing/maintaining co-worker social support (*r* = 0.18, *p* < 0.01), feedback seeking (*r* = 0.31, *p* < 0.01), seeking/performing tasks that energize (*r* = 0.44, *p* < 0.01), increasing/maintaining home autonomy (*r* = 0.12, *p* < 0.05), increasing/maintaining home social support (*r* = 0.14, *p* < 0.05), improving/maintaining psychological wellbeing(*r* = 0.41, *p* < 0.01). One of the proactive burnout behaviors, reducing work-home conflict, showed a significant negative relationship with proactive personality (*r* = −0.11, *p* < 0.05), and the remaining three (reducing hindering job demands, engaging in relaxing activities, and improving/maintaining physical health) showed no significant relation with proactive personality (*p* > 0.05). Convergent validity was thus partially confirmed.

Discriminant validity was almost completely supported, since eleven of the 12 proactive burnout prevention behaviors showed no significant relationship with generalized compliance (*p* > 0.05), Reducing hindering job demands was the only proactive burnout prevention behavior that showed a significant (negative) relationship with generalized compliance (*r* = −0.15, *p* < 0.01).

Nine of the 12 proactive burnout prevention behaviors at T1 showed a significant negative effect on burnout at T2: increasing/maintaining job control (*r* = −0.36, *p* < 0.01), increasing/maintaining supervisor social support (*r* = −0.14, *p* = 0.05), increasing/maintaining co-worker social support (*r* = −0.21, *p* < 0.01), seeking/performing tasks that energize (*r* = −0.23, *p* < 0.01), increasing/maintaining home autonomy (*r* = −0.28, *p* < 0.01), engaging in relaxing activities (*r* = −0.31, *p* < 0.01), reducing work-home conflict (*r* = −0.17, *p* < 0.05), improving/maintaining physical health (*r* = −0.17, *p* < 0.05), and improving/maintaining psychological wellbeing(*r* = −0.40, *p* < 0.01). The remaining three (feedback seeking, reducing hindering job demands, and increasing/maintaining home social support at T1) showed no significant relationship with burnout at T2 (*p* > 0.05).

Regarding work engagement, nine of the 12 proactive burnout prevention behaviors at T1 showed a significant positive effect on work engagement at T2: increasing/maintaining job control (*r* = 0.19, *p* < 0.01), increasing/maintaining supervisor social control (*r* = 0.38, *p* < 0.01), increasing/maintaining co-worker social support (*r* = 0.32, *p* < 0.01), feedback seeking (*r* = 0.29, *p* < 0.01), seeking/performing tasks that energize (*r* = 0.44, *p* < 0.01), engaging in relaxing activities (*r* = 0.14, *p* < 0.05), increasing/maintaining home social support (*r* = 0.19, *p* < 0.01), improving/maintaining physical health (*r* = 0.21, *p* < 0.01), improving/maintaining psychological wellbeing(*r* = 0.41, *p* < 0.01). Reducing hindering job demands, increasing/maintaining home autonomy, and reducing work-home conflict showed no significant relationship with work engagement (*p* > 0.05). Together, 11 of the 12 proactive burnout prevention behaviors showed a beneficial effect on wellbeing, resulting in reduced levels of burnout and/or increased work engagement. The only exception was reducing hindering job demands, which showed no significant effect on burnout and work engagement. Predictive validity was thus largely supported.

In sum, the exploration of the broader nomological network of proactive burnout prevention behaviors by examining its convergent, discriminant and predictive validity showed promising results, as expected relationships were confirmed for most behaviors.

## 4. Discussion

The aim of this study was to develop an inventory to assess employees’ proactive burnout prevention behaviors and explore its factorial validity and broader nomological network. The results showed that the inventory has adequate psychometric characteristics. Proactive burnout prevention captures 12 specific proactive behaviors employees can deploy in order to avoid burnout: increasing/maintaining job control, increasing/maintaining supervisor social support, increasing/maintaining co-worker social support, feedback seeking, seeking/performing tasks that energize, reducing hindering job demands, increasing/maintaining home autonomy, increasing/maintaining home social support, reducing work-home conflict, improving/maintaining physical health, improving/maintaining psychological wellbeing, and engaging in relaxing activities. The 12-factor structure was supported, indicating that aforementioned proactive burnout prevention behaviors can be regarded as specific proactive behaviors to prevent burnout. The subscales all showed sufficient internal consistency [39].

Convergent validity was partly confirmed, as eight of the 12 proactive burnout prevention behaviors showed a positive relationship with proactive personality. Contrary to our expectations, reducing hindering job demands, engaging in relaxing activities, and improving/maintaining physical health showed no significant relationship with proactive personality, and reducing work-home conflict showed a negative relationship with proactive personality. The reason that these proactive behaviors were not positively related to proactive personality may be a difference in orientation and foci. Proactive personality has an approach orientation aimed at actively expanding or enriching the environment [17,48]. In contrast, reducing hindering job demands and reducing work-home conflict have an avoidance orientation aimed at reducing or limiting job boundaries, and improving/maintaining physical health and engaging in relaxing activities are not aimed at changing the situation, but the self.

Discriminant validity was confirmed for 11 of the 12 proactive burnout prevention behaviors that showed no relationship with generalized compliance. The only exception was reducing hindering job demands, which was found to be negatively associated with generalized compliance. Since generalized compliance is a form of extra-role behavior on top of formal job requirements it can be perceived as an increase in overall job demands [42]. It could be that employees who already experience hindering job demands and feel the need to proactively try to reduce these, do not engage in organizational citizenship behavior that may increase their (hindering) job demands.

Results of the exploration of the predictive validity were encouraging, as nine of the 12 proactive burnout prevention behaviors showed a negative effect on burnout one month later, and nine of the 12 proactive burnout prevention behaviors showed a positive effect on work engagement one month later. Opposed to our assumptions, feedback seeking, reducing hindering job demands, and increasing/maintaining home social support at T1 showed no significant negative relationship with burnout at T2, and reducing hindering job demands, increasing/maintaining home autonomy, and reducing work-home conflict at T1 showed no significant positive relationship with work engagement at T2. We offer several explanations for the fact that not all expected relationships were confirmed.

First, since proactive behaviors aimed at reducing hindering job demands did not comply with any of our expectations, these behaviors need further investigation. In their research on job crafting, Tims et al. [35,49] also found no relationship between decreasing hindering job demands and an increase in employee wellbeing. As Tims et al. [49] suggest, it may be more difficult to change job demands than job resources. Work environments consist of ‘givens’ and ‘alterables’ [50] that have been found respectively harder or easier to adapt in the short-term [51]. According to Hakanen et al. [51], job demands seem to be more like ‘givens’ and may therefore be difficult or impossible to influence by the individual employee. In addition, a review on job crafting found that studies on avoidance crafting, such as reducing hindering job demands, rarely indicated positive effects on wellbeing outcomes [17,52]. It could be that these kinds of behavior may be perceived by employees as avoiding responsibility, which may make them hesitant to report it [49].

Second, in this study a one-month interval was used to examine whether proactive burnout prevention behaviors had an effect on wellbeing outcomes. It could be that some proactive actions take more or less time to take effect. Following the reasoning described above, demands seem to be more difficult to change than resources [51], so it may take more than one month to proactively reduce demands to prevent burnout. Moreover, previous research has shown that high day-level seeking resources was associated with high day-level job autonomy, and employees who seek resources on a daily basis are more engaged [53]. The findings of this study by Petrou et al. [53] thus suggest that proactively increasing resources may take less than one month to affect wellbeing.

Third, there may be a limited (direct) spillover effect from proactive behaviors in the home domain to work outcomes, as we did not find a significant negative effect of increasing/maintaining home social support on burnout. In addition, we did not find significant positive effects of reducing work-home conflict and increasing/maintaining home autonomy on work engagement. This is surprising, since previous studies have found positive effects of home social support, reducing work-home conflict, and home autonomy on wellbeing outcomes [54,55,56]. Yet, it could be that resources generated in the home domain do not always (directly) spill over to the work domain. According to the work-home resources model [57] home resources have a positive effect on work outcomes through their gain in personal resources.

Fourth, the effect of a specific kind of proactive burnout prevention behavior may depend on other variables (e.g., leadership style, self-efficacy), which represent important boundary conditions. Contextual factors and individual characteristics can influence the use and effect of proactive behaviors [11]. For example, the effect of feedback seeking on levels of burn-out may depend on supervisors’ leadership style and the extent to which an employee experiences self-efficacy [58,59].

In sum, findings of this study show promising results regarding the psychometric characteristics of the proactive burnout prevention inventory. However, further research is needed to substantiate the antecedents, outcomes and boundary conditions of proactive burnout prevention behaviors.

### 4.1. Limitations

A number of limitations regarding the present study need to be considered. First, the results were based on self-reports, which can cause method bias [60]. It could be valuable to include third party ratings (such as supervisor or peer ratings) of the proactive burnout prevention behaviors. However, this may in practice prove difficult, as these behaviors cover the work, home, and personal domain.

Second, although the samples used were quite heterogeneous in terms of the range of industries the participants worked in, the participants were relatively highly educated. This may limit the generalizability to employees with lower levels of education. As proactive behaviors require some degree of autonomy [11], and higher education levels are associated with higher job autonomy [61], it is possible that employees with lower levels of education may have less opportunity to engage in proactive burnout prevention behaviors. Future research should include employees with lower levels of education.

Third, the participants in this study were all Dutch and the survey was conducted in Dutch; therefore, it is unclear whether the findings can be generalized to other nationalities and cultures. In future studies, we will also use the English version (see Table 2) in order to examine whether the findings can be replicated in other countries and languages.

### 4.2. Theoretical Implications and Directions for Further Research

The findings of this study contribute to the literature on burnout prevention and proactive behavior by introducing a new inventory that enables the assessment of individual strategies employees can proactively use in the work, home and personal domain to prevent burnout. Accordingly, proactive burnout prevention behaviors may complement employer-initiated burnout prevention interventions to enhance the overall effectiveness of burnout intervention programs [3].

As the results of this study indicated, not all proactive burnout prevention behaviors may be effective in preventing burnout under all circumstances, suggesting several routes for further investigation. First, it may be that the initial source of strain influences the use and the effect of a specific kind of proactive burnout prevention behavior. For instance, research results indicate that emotional demands at work or at home can be more effectively alleviated by proactively increasing/maintaining home autonomy, than by increasing/maintaining home social support [55]. Future longitudinal research is needed to examine the relationship between proactive burnout prevention behaviors, different types of job and home demands and resources, and burnout.

Second, the initial level of demands and resources and expected success rate of the proactive behaviors may influence whether an employee engages in a specific kind of proactive burnout prevention behavior. If for example employees already experience a high level of support from family/friends, they may not deem it useful to try to proactively try to (further) increase home social support to prevent burnout. Moreover, an employee may not try to increase/maintain supervisor support if the supervisor has not been supportive in the past. Further research is needed into the antecedents of the proactive burnout prevention behaviors.

Third, the perceived risk of burnout may influence whether or not employees are inclined and capable to engage in specific kinds of proactive burnout prevention behaviors and whether or not these behaviors are effective. Burnout develops gradually over time as consequence of a resource depletion process caused by prolonged exposure to stressful conditions [6]. It is therefore important to establish at what stage employees become aware that they may be at risk of burnout and it is still possible to proactively intervene to avoid burnout [62]. If employees do not perceive any risk of burnout, they may not feel the need to take proactive action to prevent it, if on the other hand their perceived risk of burnout is too high, their resources may be depleted to an extend that they are no longer capable to engage in proactive behaviors. Because of this assumed non-linear relationship between proactive burnout prevention behaviors and burnout, it is important to investigate the ability and probability of employees to deploy proactive burnout prevention behaviors among participants in different stages of burnout.

Fourth, the proactive burnout prevention inventory includes proactive behaviors that show overlap with the concept of job crafting as elaborated by Tims and Bakker [63], i.e., it captures job crafting dimensions such as increasing social job resources and job challenges and decreasing hindering job demands. However, the proactive burnout prevention inventory also comprises behaviors beyond the work environment, such as increasing/maintaining home autonomy and increasing personal resources [18], and is therefore broader in scope.

### 4.3. Practical Implications

This study highlights that employees themselves can take initiative to prevent burnout. The newly developed proactive burnout prevention inventory enables the assessment of the proactive behaviors employees can engage in to prevent burnout. These behaviors are not restricted to the workplace, but include the home and personal domain as well, since factors beyond the work environment have been found to also influence the risk of burnout (e.g., [54,55]). The inventory can be used by organizations and individual employees to assess the collective or individual deployment of proactive behaviors to prevent burnout in relation to the risk of burnout, and to assess opportunities for improvement. This may increase awareness of the risk of burnout and stimulate employees to think about ways they could take proactive action to prevent burnout. However, this does not imply that burnout prevention is the sole responsibility of the employee. Since proactive behaviors are influenced by individual as well as situational factors, it is imperative that the employer facilitates a work environment in which employees feel encouraged and safe to engage in these behaviors [11].

## 5. Conclusion

Proactive behaviors of employees in the work, home and personal domain may be essential to complement organizations’ interventions to optimize the effectiveness of burnout prevention. Until now, what employees can do themselves to prevent burnout has received less research attention. The proactive burnout prevention inventory developed in this study showed adequate psychometric properties and consequently contributes to the assessment of employees’ proactive burnout prevention. If future research indeed demonstrates the effectiveness of employees’ proactive burnout prevention behaviors, an intervention to promote these behaviors can be developed and evaluated.

## Figures and Tables

**Table 1:** Proactive Burnout Prevention Behaviors: Domains, Focus Areas, Source and Number of Items.

Proactive Behavior	Domain	Focus Areas	Items Based on/Adapted From	# Items
	**Work**			
Increasing/maintaining job control		Increasing/maintaining job resources	[21]	5
Increasing/maintaining supervisor social support			[22]	3
Increasing/maintaining co-worker social support			[22,23,24]	5
Seeking feedback			[25]	3
Seeking/performing tasks that energize			[26]	4
Reducing hindering job demands		Reducing hindering job demands	[18,27,28]	8
	**Home**			
Increasing/maintaining home autonomy		Increasing/maintaining home resources	[29,30]	3
Increasing/maintaining home social support			[23,31]	5
Reducing work-home conflict		Reducing work-home conflict	[18,32,33]	7
	**Person**			
Improving/maintaining physical health Improving/maintaining psychological wellbeing		Increasing/maintaining personal resources	[18] [18]	4 6
Engaging in relaxing activities			[32]	3

**Table 2:** Proactive Burnout Prevention Inventory: Items, Means, Standard Deviations, Cronbach’s Alpha, Item-Factor Loadings and Factor Intercorrelations.

Proactive Behaviors and Constituent Items					Factor											
		*MD*	*SD*	*α*	1	2	3	4	5	6	7	8	9	10	11	12
*Increasing/maintaining co-worker social support*		3.58	0.73	0.87												
	I ask my co-workers for help, if necessary	3.80	0.82		**0.87**	0.03	0.05	−0.02	−0.13	−0.04	0.08	0.00	0.09	−0.08	−0.11	0.02
	I ask my co-workers to take over work from me, if necessary	2.98	1.00		**0.86**	−0.20	−0.07	0.10	0.01	0.13	0.02	0.02	−0.10	−0.15	0.13	0.03
	I ask my co-workers for advice, if necessary	3.80	0.79		**0.82**	−0.02	0.07	−0.10	0.03	0.02	0.17	0.03	0.06	−0.03	−0.14	−0.07
	I ask my co-workers for their opinion about a problem concerning work, if necessary	3.73	0.83		**0.79**	−0.08	0.05	−0.02	0.11	−0.08	0.08	−0.20	0.14	0.10	−0.11	0.04
*Increasing/maintaining home autonomy*		3.86	0.70	0.88	0.19											
	I make sure that I can decide for myself how to do things in my spare time	3.93	0.78		−0.07	**0.87**	−0.07	0.05	0.03	0.22	0.14	−0.07	−0.03	−0.07	0.03	−0.07
	I make sure that I am in control of how I spend my free time	3.83	0.74		−0.08	**0.83**	−0.01	0.06	−0.06	0.23	0.07	0.12	0.07	−0.12	−0.08	0.03
	I make sure that I can organize my free time myself	3.81	0.81		−0.06	**0.81**	−0.02	0.09	−0.05	0.24	0.17	−0.06	−0.09	0.00	−0.01	−0.11
*Reducing work-home conflict*		3.01	0.89	0.90	0.22	0.25										
	I make sure that I forget about work after hours	3.06	0.95		0.05	−0.09	**0.96**	0.01	−0.02	0.02	0.09	0.04	−0.07	−0.07	−0.04	−0.05
	I make sure that I distance myself from work after hours	3.32	0.98		−0.01	−0.04	**0.90**	−0.02	−0.01	0.01		−0.02	−0.02	0.02	−0.06	0.07
	I make sure that I don’t think about work at all after hours	2.63	1.00		0.06	0.03	**0.86**	−0.02	−0.02	0.06	−0.07	−0.00	0.04	−0.02	0.05	−0.03
*Increasing/maintaining job control*		3.90	0.52	0.80	0.12	0.31	0.19									
	I make sure that I am in control of how I carry out my work	4.11	0.53		−0.16	0.08	−0.08	**0.84**	−001	−0.01	0.16	0.04	0.05	−0.22	−0.12	−0.01
	I make sure that I am in control of when I carry out my work	3.84	0.79		0.05	−0.06	0.02	**0.79**	0.03	−0.06	−0.09	−0.07	−0.04	0.07	0.16	−0.04
	I make sure that I am in control of my work	4.05	0.58		−0.16	0.18	0.04	**0.67**	0.06	−0.17	−0.13	0.09	0.11	0.06	−0.26	0.09
	I make sure that I am in control of the pace at which I carry out my work	3.75	0.80		0.24	0.10	−0.02	**0.62**	0.03	−0.02	−0.18	0.01	0.03	0.02	0.13	0.02
	I make sure that I am in control of my workload	3.75	0.76		0.23	0.18	0.13	**0.36**	−0.12	0.10	−0.23	0.08	−0.20	0.30	0.15	−0.06
*Improving/maintaining psychological wellbeing*		3.68	0.65	0.82	0.19	0.25	0.20	0.28								
	I try to view stressful situations from different angles	3.64	0.81		−0.05	0.06	−0.05	0.14	**0.83**	−0.12	0.11	−0.18	0.05	0.10	−0.06	0.03
	I try to put stressful situations into perspective	3.67	0.83		−0.12	−0.04	0.12	−0.00	**0.79**	−0.06	0.11	−0.07	−0.03	0.25	−0.05	0.07
	I stimulate a positive mindset in myself	3.66	0.84		0.07	−0.23	−0.06	0.08	**0.77**	0.24	−0.07	0.20	−0.09	−0.18	0.05	−0.06
	I try to approach a problem positively	3.76	0.73		0.12	0.09	−0.07	−0.14	**0.75**	0.14	−0.10	0.13	0.11	−0.13	0.02	−0.13
*Engaging in relaxing activities*		3.57	0.76	0.92	0.05	0.15	0.29	0.09	0.18							
	I make sure that I do relaxing things after work	3.56	0.80		−0.00	0.28	−0.02	−0.06	−0.01	**0.82**	−.001	0.06	0.01	0.02	0.02	0.11
	I makes sure that I kick back and relax after work	3.64	0.80		−0.02	0.25	0.06	−0.10	0.08	**0.80**	−0.16	−0.09	0.12	0.14	0.07	0.04
	I make sure that I take time for relaxing activities after work	3.50	0.84		0.07	0.27	0.03	−0.02	0.04	**0.78**	−0.04	0.08	−0.05	0.07	−0.08	0.09
*Increasing/maintaining home social support*		3.20	0.85	0.85	0.28	−0.04	0.15	0.00	0.07	0.23						
	I ask my family/friends for their opinion about a problem (concerning work), if necessary	3.15	1.08		−0.02	0.08	−0.00	−0.10	−0.11	−0.08	**0.80**	0.14	0.09	0.10	0.10	0.03
	I ask my family/friends for advice, if necessary	3.35	0.93		0.22	0.20	0.05	−0.08	0.05	−0.10	**0.79**	0.05	−0.03	0.05	−0.04	−0.01
	I ask my family/friends for help, if necessary	3.10	0.89		0.19	0.16	−0.01	0.03	0.16	−0.01	**0.76**	−0.08	−0.19	0.05	0.09	0.05
*Seeking/performing tasks that energize*		3.85	0.66	0.84	0.23	0.24	0.18	0.38	0.36	0.16	0.11					
	I actively take on tasks that enable me to develop myself further	3.82	0.79		−0.11	−0.23	−0.02	−0.06	−0.00	0.02	−0.02	**0.95**	−0.01	0.08	0.09	0.00
	I consciously take on challenging tasks that give me energy	3.82	0.78		−0.01	−0.02	0.08	0.04	0.02	0.03	0.04	**0.85**	0.05	−0.04	−0.11	−0.02
	I apply for tasks that may be instructive to me	3.93	0.73		0.05	0.03	−0.08	0.17	−0.04	−0.01	0.29	**0.64**	−0.04	0.15	−0.080	0.06
*Increasing/maintaining supervisor social support*		3.61	0.80	0.86	0.35	0.02	0.07	0.17	0.09	0.03	0.31	0.27				
	I actively try to build a good relationship with my supervisor	3.75	0.83		−0.07	0.03	0.02	−0.05	0.13		−0.13	−0.00	**0.91**	0.04	−0.01	0.05
	I ask my supervisor for support, if necessary	3.54	0.96		0.21		−0.03	0.09	−0.08	0.07	−0.03	−0.04	**0.78**	0.07	0.09	−0.04
	I ask my supervisor for his/her opinion about a problem concerning work, if necessary	3.55	0.93		0.21	−0.14	−0.08	0.11	−0.07	0.04	0.14	0.08	**0.68**	0.04	0.06	−0.02
*Feedback seeking*		2.64	0.76	0.70	0.41	0.14	0.09	0.26	0.27	−0.08	0.12	0.36	0.42			
	I seek feedback from my supervisor about my work performance	2.77	0.96		−0.16	−0.13	−0.05	0.07	0.03	0.10	0.15	−0.02	0.11	**0.82**	0.01	−0.02
	I seek information from co-workers about my work performance	2.68	0.83		−0.00	−0.23	−0.03	0.00	0.02	0.11	0.18	−0.03	−0.03	**0.82**	0.13	−0.02
	I seek feedback from my supervisor about potential advancement within my organization	2.48	1.08		−0.02	0.15	−0.01	−0.16	0.01	0.02	−0.12	0.22	0.08	**0.62**	−0.17	−0.09
*Reducing hindering job demands*		2.80	0.69	0.73	0.33	0.29	0.29	0.29	0.20	0.14	0.03	0.14	0.09	0.16		
	I make sure that I do not have to carry out tasks that cost too much energy	2.57	0.89		−0.04	−0.14	−0.07	−0.04	0.02	0.01	−0.06	−0.05	−0.02	0.09	**0.91**	0.10
	I make sure that my work is the least burdening/straining	2.62	0.81		−0.14	0.09	−0.00	0.01	−0.10	0.03	0.23	−0.02	0.10	−0.09	**0.87**	−0.12
	I organize my work so I don’t get too stressed out	3.21	0.86		−0.12	0.33	0.27	0.02	0.17	−0.22	0.05	0.15	0.14	−0.13	**0.48**	0.09
*Improving/maintaining physical health*		3.67	0.72	0.71	0.04	0.16	0.32	−0.01	0.03	0.20	0.08	0.11	0.00	0.01	0.06	
	I make sure that I get enough exercise	3.71	0.87		−0.05	−0.22	0.09	0.08	0.03	0.19	0.10	−0.06	0.04	−0.08	−0.00	**0.82**
	I make sure that I engage enough in sports	3.35	1.14		−0.08	−0.13	0.07	0.02	−0.10	0.27	0.05	0.02	0.05	−0.04	−0.04	**0.77**
	I make sure I eat and drink healthy	3.94	0.65		0.22	0.30	−0.21	−0.14	0.01	−0.20	−0.10	0.09	−0.08	−0.00	0.10	**0.70**

*Note*. N = 142; answers are provided on a 5-point Likert type scale (1 = never; 5 = always); Item-Factor Loadings as derived from an Exploratory Factor Analysis (PCA) with Promax Rotation.

**Table 3:** Means, Standard Deviations, Cronbach’s alphas, and Correlations between Proactive Burnout Prevention Behaviors and Proactive Personality, Generalized Compliance, Burnout, and Work Engagement.

		*M*	*SD*	*α*	1	2	3	4	5	6	7	8	9	10	11	12
1	Increasing/maintaining job control (T1)	3.93	0.49	0.78	--											
2	Increasing/maintaining supervisor social support (T1)	3.57	0.77	0.82	**0.13** *	--										
3	Increasing/maintaining co-worker social support (T1)	3.49	0.72	0.86	0.08	**0.52** **	--									
4	Feedback seeking (T1)	2.60	0.75	0.72	**0.14** *	**0.45** **	**0.33** **	--								
5	Seeking/performing tasks that energize (T1)	3.80	0.65	0.83	**0.25** **	**0.32** **	**0.22** **	**0.39** **	--							
6	Reducing hindering job demands (T1)	2.83	0.72	0.76	**0.34** **	0.03	0.00	−0.09	−0.05	--						
7	Increasing/maintaining home autonomy (T1)	3.87	0.70	0.89	**0.32** **	−0.02	0.05	−0.05	0.05	**0.28** **	--					
8	Engaging in relaxing activities (T1)	3.56	0.71	0.90	**0.21** **	0.04	0.10	−0.00	**0.12** *	**0.26** **	**0.47** **	--				
9	Increasing/maintaining home social support (T1)	3.12	0.87	0.87	0.07	**0.27** **	**0.38** **	**0.29** **	**0.18** **	0.10	**0.19** **	**0.19** **	--			
10	Reducing work-home conflict (T1)	3.02	0.91	0.90	**0.20** **	0.04	0.09	−0.03	−0.02	**0.29** **	**0.18** **	**0.31** **	0.05	--		
11	Improving/maintaining physical health (T1)	3.64	0.75	0.73	0.05	0.07	0.09	0.05	**0.14** **	0.10	**0.12** *	**0.31** *	**0.15** **	**0.17** **	--	
12	Improving/maintaining psychological wellbeing(T1)	3.69	0.65	0.84	**0.23** **	**0.22** **	**0.21** **	**0.22** **	**0.38** **	0.06	**0.11** *	**0.20** **	0.09	0.11	0.08	--
13	Proactive Personality (T1)	3.61	0.61	0.79	**0.18** **	**0.27** **	**0.18** **	**0.31** **	**0.44** **	−0.07	**0.12** *	0.01	**0.14** *	−**0.11** *	0.05	**0.41** **
14	Generalized Compliance (T1)	4.28	0.62	0.76	0.05	0.05	−0.06	−0.00	0.08	−**0.15** **	0.06	−0.01	−0.09	−0.06	−0.01	0.01
15	Burnout (T2)	2.08	0.56	0.94	−**0.36** **	−**0.14** *	−**0.21** **	−0.06	−**0.23** **	−0.01	−**0.28** **	−**0.31** **	−0.11	−**0.17** *	−**0.17** *	−**0.40** **
16	Work Engagement (T2)	4.04	1.22	0.95	**0.19** **	**0.38** **	**0.32** **	**0.29** **	**0.44** **	−0.12	0.10	**0.14** *	**0.19** **	−0.04	**0.21** **	**0.41** **

*Note*. *N* = 343 for (T1) intercorrelations and *N* = 201 for (T1-T2) intercorrelations. ** *p* < 0.01, * *p* ≤ 0.05 *p*-values were not corrected for multiple testing. Results of correlations < 0.30 are exploratory significant.

## Supplementary Materials

The following is available online at https://www.mdpi.com/1660-4601/17/5/1711/s1.
