# Supplementary material for: The Development of a Proactive Burnout Prevention Inventory: How Employees Can Contribute to Reduce Burnout Risks
Source: Int J Environ Res Public Health. 2020 Mar 5;17(5):1711. doi: 10.3390/ijerph17051711 (PMC7084396; doi:10.3390/ijerph17051711)
Supplement: Supplementary file 1 [file ijerph-17-01711-s001.pdf]

Supplementary Material

The Development of a Proactive Burnout Prevention Inventory: How Employees Can Contribute to Reduce Burnout Risks

Madelon C. B. Otto*, Joris Van Ruysseveldt, Nicole Hoefsmit and Karen Van Dam

Department of Psychology, Open University of the Netherlands, 6419 AT Heerlen, Netherlands; joris.vanruysseveldt@ou.nl (J.v.R.); nicole.hoefsmit@ou.nl (N.H.); karen.vandam@ou.nl (K.v.D.)

* Correspondence: madelon.otto@ou.nl (M.C.B.O.)

Description of Proactive Burnout Prevention Behaviors

Proactive burnout prevention captures 12 specific proactive behaviors that cover work-, home- and person-oriented actions, and aim at both demands and resources.

1. Increasing/maintaining job control

Participants in the ESP reported that making sure one is able to control when, how, and what to do at work is an important resource to prevent burnout [1]. Since Karasek [2] introduced the job demand-control (JDC) model in 1979, job control has received extensive research attention as a crucial factor in the prevention of stress and burnout [3]. Ample research confirms that a lack of job control is an important determinant of burnout [e.g., 4,5], suggesting that proactively ensuring sufficient job control can prevent burnout.

2 and 3. Increasing/maintaining supervisor and/or increasing/maintaining co-worker social support

Study findings indicated that employees ask their supervisors and/or co-workers for advice and help in order to deal with (high) demands to avoid burnout [1]. Social support may in this case involve emotional, instrumental, informational, and appraisal support from supervisors and co-workers [e.g., 3,6,7]. Social support has been added to the JDC model [2] in the 1980s [8], and has since also been extensively researched as another key job resource in the prevention of burnout [e.g., 9,10]. Taking proactive action to increase and/or maintain social support at work is therefore expected to help prevent burnout.

4. Feedback seeking

Research results showed that employees engage in self-initiated anticipatory action by asking their supervisor or co-workers for feedback on their functioning in order to reduce feelings of distress [1]. Various studies have shown evidence that (performance) feedback is a job resource that can influence burnout [e.g., 11,12], indicating that feedback seeking may be a useful strategy to prevent burnout.

5. Seeking or performing tasks that energize

Participants in the ESP mentioned that they actively engage in or ask for tasks that energize them to prevent burnout [1]. These tasks seem to trigger positive emotions (e.g., eagerness, excitement) and therefore show similarities with challenging demands as described by Crawford, LePine, and Rich [13]. Crawford et al. [13] explain the distinction between hindering job demands (e.g., hassles, role overload) that have been found to be related to negative outcomes and challenging job demands (e.g., task complexity) that have been found to be related to positive outcomes. Challenging demands are considered opportunities for personal growth and future gains [13]. Studies have found negative relationships between crafting challenging job demands and levels of burnout [e.g., 14,15], suggesting that actively seeking or performing tasks that energize may actually replenish resources in order to prevent burnout.

6. Reducing hindering job demands

Study findings revealed that employees attempt to reduce their hindering job demands by organizing their work in such a way, that it does not deplete their energy and resources [1]. Job

demands have since long been identified as key factors for the development of burnout [e.g., 2,16]. Specifically, hindering job demands have been associated with higher levels of burnout [17]. It is therefore expected that proactively reducing hindering job demands can prevent burnout.

7. Increasing/maintaining home autonomy

Participants in the ESP reported that it is important for employees to not have too many obligations after work, in order to perceive some flexibility in how to spend their free time [1]. Study results have shown a negative relationship between home autonomy and burnout [18]. Ensuring one is able to determine autonomously how to spend one's leisure time is thus expected to conserve home resources and help prevent burnout.

8. Increasing/maintaining home social support

Employees indicated to ask advice or help from family and/or friends to deal with stressful conditions [1]. In addition to the extensive research evidence that demonstrates that workplace social support is an important factor in the prevention of burnout, home social support has also been found to decrease levels of burnout [e.g., 19,20]. These findings suggest that ensuring social support from family and/or friends can help employees in the prevention of resource depletion and burnout.

9. Reducing work-home conflict

Study findings showed that feelings of exhaustion reported by participants were not only caused by job demands, but stemmed from a combination of (high) job demands and duties and requirements at home (e.g., having to take care of a family members, dealing with a stressful life event) [1]. They therefore stated to proactively try to reduce work-home conflict in order to retain resources to prevent burnout [1]. The proactive actions employees indicated to undertake involved distancing themselves from work, for instance, by not answering work related phones and e-mails after working hours, and by reducing task-related demands at home, such as delegating and/or reducing household duties [1]. Reducing work-home conflict has been negatively linked to burnout [e.g., 20], so proactively reducing work-home conflict is assumed to help prevent burnout.

10. Improving/maintaining physical health

Findings of the ESP suggested that employees proactively try to engage in physical activity and adopt a healthy lifestyle to avoid resource depletion [1]. Research confirms that physical health (e.g., exercise, healthy lifestyle) is negatively related to burnout [e.g., 21,22] and thus taking proactive actions to maintain or improve their physical health is expected to help employees to prevent burnout.

11. Improving/maintaining psychological wellbeing

Employees indicated to proactively engage in actions to improve and/or maintain their psychological wellbeing [1]. This included creating a positive mindset, cognitively reappraising stress provoking thoughts or situations, and actively engaging in mindfulness activities to create peace of mind [1]. Studies have shown that psychological wellbeing and burnout are negatively related [23,24], suggesting that undertaking proactive action to conserve psychological resources can aid in avoiding burnout.

12. Engaging in relaxing activities

Participants in the ESP stated to consciously schedule moments for relaxation after working hours to replenish their personal resources in order to prevent burnout [1]. In the literature, relaxation is considered a type of recovery from work behavior that can result in reduced levels of burnout [25]. Actively taking time for relaxing (activities) may therefore help to prevent burnout.

References

1. Otto, M.C.B.; Hoefsmit, N.; Van Ruysseveldt, J.; Van Dam, K. Exploring proactive behaviors of employees in the prevention of burnout. *International journal of environmental research and public health* **2019**, *16*, doi:10.3390/ijerph16203849.

2. Karasek, R.A. Job demands, job control, and mental strain: Implications for job redesign. *Administrative Science Quarterly* **1979**, *24*, 285-308.
3. Van der Doef, M.; Maes, S. The job demand-control (-support) model and psychological well-being: a review of 20 years of empirical research. *Work & Stress* **1999**, *13*, 87-114, doi:10.1080/026783799296084.
4. Park, H.I.; Jacob, A.C.; Wagner, S.H.; Baiden, M. Job control and burnout: A meta-analytic test of the conservation of resources model. *Applied Psychology: An International Review* **2014**, *63*, 607-642, doi:10.1111/apps.12008.
5. Day, A.; Crown, S.N.; Ivany, M. Organisational change and employee burnout: The moderating effects of support and job control. *Safety Science* **2017**, *100*, 4-12, doi:10.1016/j.ssci.2017.03.004.
6. House, J.S. *Work stress and social support*; Addison-Wesley Pub. Co.: Reading, Mass., 1981.
7. Kumar, A.; Channa, K.; Bhutto, N. When and how workplace social support improves family performance. *Applied Research in Quality of Life* **2019**, 1-22.
8. Johnson, J.V.; Hall, E.M. Job strain, work place social support, and cardiovascular disease: a cross-sectional study of a random sample of the Swedish working population. *The American Journal of Public Health* **1988**, *78*, 1336-1342.
9. Karasek, R.A. Demand-control model: A social-emotional, and psychological approach to stress risk and active behavior development. In *Encyclopedia of occupational health and safety*, 4th ed.; International Labour Office: Geneva, 1998; pp. 34.36-34.14.
10. Hausser, J.A.; Mojzisch, A.; Niesel, M.; Schulz-Hardt, S. Ten years on: A review of recent research on the job demand-control (-support) model and psychological well-being. *Work & Stress* **2010**, *24*, 1-35.
11. Borden, L.; Levy, P.; Silverman, S. Leader arrogance and subordinate outcomes: The role of feedback processes. *Journal of Business and Psychology* **2018**, *33*, 345-364.
12. Gong, Z.; Zhang, J.; Zhao, Y.; Yin, L. The relationship between feedback environment, feedback orientation, psychological empowerment and burnout among police in China. *Policing: An International Journal of Police Strategies & Management* **2017**, *40*, 336-350.
13. Crawford, E.R.; LePine, J.A.; Rich, B.L. Linking job demands and resources to employee engagement and burnout: A theoretical extension and meta-analytic test. *Journal of Applied Psychology* **2010**, *95*, 834-848, doi:10.1037/a0019364.
14. Tims, M.; Bakker, A.B.; Derks, D. The impact of job crafting on job demands, job resources, and well-being. *Journal of Occupational Health Psychology* **2013**, *18*, 230-240, doi:10.1037/a0032141.
15. Petrou, P.; Demerouti, E.; Schaufeli, W.B. Job crafting in changing organizations: Antecedents and implications for exhaustion and performance. *Journal of Occupational Health Psychology* **2015**, *20*, 470-480, doi:10.1037/a0039003.
16. Bakker, A.B.; Demerouti, E. Job demands–resources theory: Taking stock and looking forward. *Journal of Occupational Health Psychology* **2017**, *22*, 273-285, doi:10.1037/ocp0000056.
17. Van den Broeck, A.; De Cuyper, N.; De Witte, H.; Vansteenkiste, M. Not all job demands are equal: Differentiating job hindrances and job challenges in the job demands-resources model. *European Journal of Work and Organizational Psychology* **2010**, *19*, 735-759, doi:10.1080/13594320903223839.
18. Bakker, A.B.; Demerouti, E.; Schaufeli, W.B. The crossover of burnout and work engagement among working couples. *Human Relations* **2005**, *58*, 661-689, doi:10.1177/0018726705055967.
19. Peeters, M.C.W.; Le Blanc, P.M. Towards a match between job demands and sources of social support: A study among oncology care providers. *European Journal of Work & Organizational Psychology* **2001**, *10*, 53-72, doi:10.1080/13594320042000034.
20. Verweij, H.; Van der Heijden, F.M.M.A.; Van Hooff, M.L.M.; Prins, J.T.; Lagro-Janssen, A.L.M.; Van Ravesteijn, H.; Speckens, A.E.M. The contribution of work characteristics, home characteristics and gender to burnout in medical residents. *Advances in Health Sciences Education* **2017**, *22*, 803-818.
21. Lease, S.H.; Ingram, C.L.; Brown, E.L. Stress and health outcomes: Do meaningful work and physical activity help? *Journal of Career Development* **2019**, *46*, 251-264.
22. Jones III, A.; Norman, C.S.; Wier, B. Healthy lifestyle as a coping mechanism for role stress in public accounting. *Behavioral Research in Accounting* **2010**, *22*, 21-41, doi:10.2308/bria.2010.22.1.21.
23. Wright, T.A.; Hobfoll, S.E. Commitment, psychological well-being and job performance: An examination of conservation of resources (COR) theory and job burnout. *Journal of Business & Management* **2004**, *9*, 389-406.

24. Salvagioni, D.A.J.; Melanda, F.N.; Mesas, A.E.; González, A.D.; Gabani, F.L.; Andrade, S.M.d. Physical, psychological and occupational consequences of job burnout: A systematic review of prospective studies. *PLoS ONE* **2017**, *12*, 1-29, doi:10.1371/journal.pone.0185781.
25. Sonnentag, S.; Venz, L.; Casper, A. Advances in recovery research: What have we learned? What should be done next? *Journal of Occupational Health Psychology* **2017**, *22*, 365-380, doi:10.1037/ocp0000079.
